# Internet and Pornography Use During the COVID-19 Pandemic: Presumed Impact and What Can Be Done

**DOI:** 10.3389/fpsyt.2021.623508

**Published:** 2021-03-16

**Authors:** Hashir Ali Awan, Alifiya Aamir, Mufaddal Najmuddin Diwan, Irfan Ullah, Victor Pereira-Sanchez, Rodrigo Ramalho, Laura Orsolini, Renato de Filippis, Margaret Isioma Ojeahere, Ramdas Ransing, Aftab Karmali Vadsaria, Sanya Virani

**Affiliations:** ^1^Dow University of Health Sciences, Karachi, Pakistan; ^2^Kabir Medical College, Gandhara University, Peshawar, Pakistan; ^3^Department of Child and Adolescent Psychiatry, School of Medicine, New York University, New York, NY, United States; ^4^Department of Social and Community Health, School of Population Health, University of Auckland, Auckland, New Zealand; ^5^Unit of Clinical Psychiatry, Department of Neurosciences/DIMSC, School of Medicine and Surgery, Polytechnic University of Marche, Ancona, Italy; ^6^Psychiatry Unit, Department of Health Sciences, University Magna Graecia of Catanzaro, Catanzaro, Italy; ^7^Department of Psychiatry, Jos University Teaching Hospital, Jos, Nigeria; ^8^Bhaktshreshtha Kamalakarpant Laxman Walawalkar Rural Medical College, Kasarwadi, India; ^9^Smt. Kashibai Navale Medical College and General Hospital, Pune, India; ^10^Veterans Affairs Connecticut Healthcare System, Yale University School of Medicine, West Haven, CT, United States; ^11^School of Medicine Yale University, New Haven, CT, United States

**Keywords:** COVID-19, problematic internet use, pornography, behavioral addictions, mental health

## Abstract

The COVID-19 pandemic continues to cause an immense psychosocial strain worldwide. Excessive use of the internet during these psychologically trying times, fueled by physical isolation as a result of lockdowns, has translated into dysfunctional behaviors. A growing body of evidence suggests an unprecedented increase in internet use and consumption of online pornography during the pandemic, and possibly even directly caused by it. In this review, the authors report data from relevant sources to show the rise in pornography use during lockdowns in different countries worldwide. In addition to a brief overview of the neurobiology of internet addiction broadly and problematic online pornography use specifically, similarities with substance use disorders are explained. Further, the current status of the debate about defining diagnostic criteria is discussed. Finally, the review sheds light on the potential detrimental outcomes during the future post-pandemic “re-adaptation,” while simultaneously offering preventative and management strategies for harm reduction. The authors conclude that foresightedness with utilizing existing tools and therapies and exercising appropriate amounts of caution could go a long way in addressing the challenges that lie ahead in the post-pandemic era.

## Introduction

Crossing a 100 million cases and with more than 2 million deaths recorded globally to date (1), the COVID-19 pandemic has transformed the world. The socioeconomic consequences have been dire, leaving many unemployed and grappling with a constant state of uncertainty and anxiety, reinforced by the tremendous amounts of “free time” they now have in the absence of jobs and the compounding isolation due to COVID-19 enforced regulations. This in turn has led to a rapid uptake of maladaptive and dysfunctional behaviors among all age groups, at the crux of which lies excessive internet consumption (2, 3).

BBC and Netflix^Ⓒ^ recorded 16 million new subscribers in the first 3 months of 2020, almost 100% higher than the new subscribers during the last few months of 2019 (4). In April, Microsoft's^Ⓒ^ game servers had 10 million users, showing how the internet gaming industry has thrived in the pandemic (5). A preliminary study in China comparing data between October 2019 and March 2020 reported a sharp increase (23%) in the prevalence of severe internet addiction with a 20-fold rise in the dependence degree of those already addicted to the internet (6). Another study conducted in China limited to adolescents depicted a rise in internet use, especially in subjects considered as “Addictive Internet Users” based on the questionnaire's cutoff (2). A cross-sectional study in Taiwan claimed that the prevalence of internet addiction in adolescents was much higher than other previously recorded samples worldwide (7).

This review summarizes viewpoints on behavioral addictions with focus on problematic internet use and pornography, elucidates what is known to date about their neurobiology, describes how the pandemic has intensified the problem by providing most current statistics, and discusses the need for diagnostic criteria, while offering strategies for prevention and harm reduction during the pandemic and post-pandemic era.

### Internet Addiction

Internet addiction, also referred to as “pathological internet use” or “problematic use of internet” (PUI), has been defined as “a psychological dependence on the internet” (8), and is characterized by excessive or poorly controlled preoccupations, urges, or behaviors regarding internet usage, leading to impairment or distress (9, 10). The need for defining a specific behavioral addiction to the internet has been a subject for debate since the early 1990s, when the first cases of internet addiction were described (11). Two discrete manifestations of PUI are (12): (a) generalized—a non-specific, multifaceted overuse of the internet, not directly related to any one activity; and (b) specific—a pathological indulgence in one (or more, but separate) activity on the internet, using internet as a medium. In a 2014 study, they were referred to as GIA (generalized internet addiction) and SIA (specific internet addiction) (13).

The use of internet addiction as an umbrella term is, hence, closely related to considering the internet as just the channel to online content. Various internet-mediated problematic behaviors have been described, including but not limited to problematic online pornography use, internet gaming disorder, online gambling, and excessive use of social media and communication sites.

### Pornography Addiction

A 2006 longitudinal study on internet addiction concluded that of the many internet-related activities, “erotica” (or online pornography) had the greatest potential to be addictive (14). According to Stein et al. in persons with Compulsive Sexual Behavior Disorder (CSBD), the behavior becomes a central focus of their life, with unsuccessful efforts to control or significantly reduce it as well as adverse consequences (e.g., repeated relationship disruption, occupational consequences, negative impact on health) (15).

Known as both a type of internet-mediated addiction and a component of hypersexuality, problematic online pornography use is rapidly turning into a topic that demands deeper empirical research due to its potentially addictive nature and perceived negative outcomes.

Despite its presumed pervasiveness, “internet pornography addiction” (IPA) or “problematic online pornography use” (POPU) is under-researched, and usually fitted into the umbrella construct of hypersexual behavior or “compulsive sexual behavior” (CSB). Some have attempted to characterize IPA/POPU as an “impulse-control disorder” while the International Classification of Diseases (ICD-11) has placed it under compulsive sexual behavior disorder (CSBD), following the impulse-control disorder model. On the contrary, the Diagnostic and Statistical Manual of the American Psychiatric Association (DSM-5) seems to follow the addiction model since IPA shares various classic characteristics (like tolerance) with other addictions. Additionally, some authors argue that there is a considerable overlap between compulsive (anxiety-reducing) behaviors and impulsive (rewarding) behaviors when it comes to IPA, despite noticeable dissimilarities. It is important to note that Stein et al. present thought-provoking arguments in favor of using the underlying mechanisms for classification rather than solely adopting a “descriptivist” approach (15).

## Neurobiology of Internet and Pornography Addiction

### Evidence Related to Internet Addiction

While behavioral factors make internet addiction clinically recognizable, neurobiological studies have to be combined with this behavioral analysis in what has been labeled “parallel and contiguous paradigms” (16). Some important studies investigating the neurobiological aspect of internet addiction have found similarities between it and pathological gambling and substance use disorders, especially in the loss of executive control (13). Negative associations of internet addiction to activity in brain areas which are core components of the default mode network (precuneus, posterior cingulate gyrus) were similar to those in other substance and behavioral addictions, and some impaired brain mechanisms in the inhibitory control network could explain the lack of control found in such behavioral addictions (17). It is hypothesized that dysfunctions in dopaminergic circuits make the individual more prone to addictive behaviors (like internet gaming or pornography) that feed reward mechanisms (18).

As with disordered gambling, the Taq1A1 allele of the DRD2 gene (19) and homozygosity of the short allelic variant of the 5-HTTLPR gene (20) have been associated with PUI.

### Neural Mechanisms of Pornography Addiction and Supranormal Stimuli

A common neurobiological stem between addiction resulting from consumption of psychoactive substances and CBSD/IPA is recognizable. Some studies have proposed commonalities between neural mechanisms of drug-related and behavior-related addictions, especially when CSBD/IPA is brought into focus (21). A malfunctioning of the brain's reward center has been suggested as being responsible for turning these behaviors into addictions (22). A significant negative association between watching more pornographic content per week and right caudate volume, and between cue-reactivity and left putamen was also found, which could be the result of a constant stimulation of the reward centers or a neuroplastic change allowing for greater pleasure while consuming pornographic content (23). Furthermore, men with problematic use of online pornography were found to have greater ventral striatal activity when predicting erotic pictures (24), concluding that this processing of cues was similar to conventional addictions (SUD) and contributed to the clinical presentation.

A peculiar addition to the neurobiology of IPA is the concept of “supranormal stimulus,” introduced in the book “The Study of Instinct” (25) published in 1951. It refers to the brain's reward systems as being activated at greater levels by an artificial (or engineered) stimulus than by a natural stimulus of a similar type. In 2010, internet pornography was added as an example illustrating the phenomenon of supranormal stimulus (26), owing to the “infinite” number of artificial scenarios available online for the consumer to choose from. This allows for the individual to seek greater reward and compulsively consume pornography, entering the “addictive mode.” This has a tie-end to novelty-seeking behavior found in people with a pornography addiction and the desire for unique, new, and more perfect content to make it a subject of masturbation/sexual desire—also called a “pathological pursuit” (27). This can also manifest in the shift from pornographic magazines to online video pornography (28). Park et al. builds upon pornography as a supranormal stimulus by highlighting the “novelty” it registers and uses case reports to explain the negative effects it may bear on a person's life because of the inability to achieve the same response in real-life as compared to person's response to pornography (29).

Of note, according to Stein et al. (15), CSBD is not considered a true compulsion that occurs in relation to intrusive, unwanted and typically anxiety-provoking thoughts (obsessions) as in OCD but a repetitive, typically initially rewarding behavior pattern that the person feels unable to control, which appears to have both impulsive and compulsive elements (30). While the earlier course is predominantly related to impulsivity and positive reinforcement, the latter is more about compulsive behaviors and negative reinforcement (31). The dual-control model posits that CSBD becomes an issue when self-control and sexual responsiveness/excitability are high and low, respectively (32).

## The Need for Diagnostic Criteria

In a post-COVID world, there is potential for mounting complaints of behavioral addictions requiring robust actions to prevent them from becoming another major public mental health problem, as substance abuse disorders already are. Accurate and holistic diagnostic patterns would need to be found before categorizing each symptom or even a slightly problematic use of internet content(s) as an addiction. Fineberg et al. included the development of diagnostic criteria as 1 of the 9 fundamental aims for their European task force to broaden the understanding of internet addiction (33). While diagnostic criteria for internet addiction have been proposed, consensus is still lacking. The most holistic criteria, which considered previous proposals and conducted a validation and clinical trials, was brought about in 2010 (34). Previously, Young's Diagnostic Questionnaire and Young's Internet Addiction Test were developed by using the criteria for diagnosis of pathological gambling or other conventional addictions as a basis (35, 36).

The current situation engenders a precedent for other, more specific types of internet-related addictions (like IPA) to be diagnosed with a precisely developed and targeted criteria by using existing models for generalized internet addiction. This is closely linked with internet addiction being viewed as a misnomer and an obsolete description by Starcevic (37). The author suggests the use of independent terms describing addictions caused by different types of content on the internet (for e.g., IPA, internet gaming disorder, etc.) instead of using just internet addiction (which is too generalized and non-specific) (37). Therefore, the need for a more wide-spectrum diagnostic criteria, especially in the backdrop of COVID-19, is rapidly becoming more and more pressing. A subjective method is needed to ascertain and diagnose the addictive aspect of specific types of content (comparable to conventional types of substances) being consumed while using the internet as a conduit. The I-PACE model (38) is one recent development that can be used as a basis to develop further screening or diagnosing methods for different types of internet addiction, or at least as a way of labeling the disorders (for e.g., based on the “first-choice” content used and/or mixed if 2 types of contents are co-dominant). This, however, will only be possible if enough empirical data is collected to ascertain the validity of this framework in clinical scenarios.

In contrast to the ICD-10 that included the category of “excessive sexual drive” without a description of symptoms but referencing “nymphomania” and “satyriasis,” the ICD-11 guidelines describe Compulsive Sexual Behavior Disorder (placed in the Mental and Behavioral Disorders chapter) as a “persistent pattern of failure to control intense, repetitive sexual impulses or urges resulting in repetitive sexual behavior” (15). However, the ICD-11 avoids focusing on etiological issues like traumatic sexual experiences that might lead an individual to use sex as a coping strategy in response to negative emotions.

## The Influence of COVID-19 and the Lockdown

During the COVID-19 imposed lockdowns across the world, the internet offered never-ending distractions for people forced to stay home. A study conducted on subjects older than 60 showed significantly increased internet use with a 64.1% rise in usage of online communication applications like Zoom/WhatsApp and a 41.7% rise in using the internet for daily errands, showing how even middle-aged subjects and older adults who were not spending a long time on the internet previously, have been quasi-forced to adopt online activities because of multiple pressures such as conversion of on-site workplaces to internet-based work-from-home environments and the need to stay updated with COVID-related news and family (39).

The COVID-19 lockdown translated into physical isolation, driving individuals to waste time online with no definite purpose, spending longer, abnormal durations of time online when bored (40), leading to increased consumption of online pornography. In 2019, Pornhub^Ⓒ^, one of the world's largest pornographic video-sharing websites, received 42 billion visits—roughly 5 times the world's population (41). But the pandemic seems to have caused an even sharper and more noticeable surge in traffic on pornographic websites. Pornhub has shared statistics on a regular basis revealing the changes and trends in the consumption of their content, showing a constantly positive deviation from average traffic on an average pre-pandemic day (42). A study employing Google Trends and joint point regression analysis demonstrated a significant rise (compared to last 4 years) in interest for pornographic websites in countries with “stay at home orders” (43).

**Figure 1:**
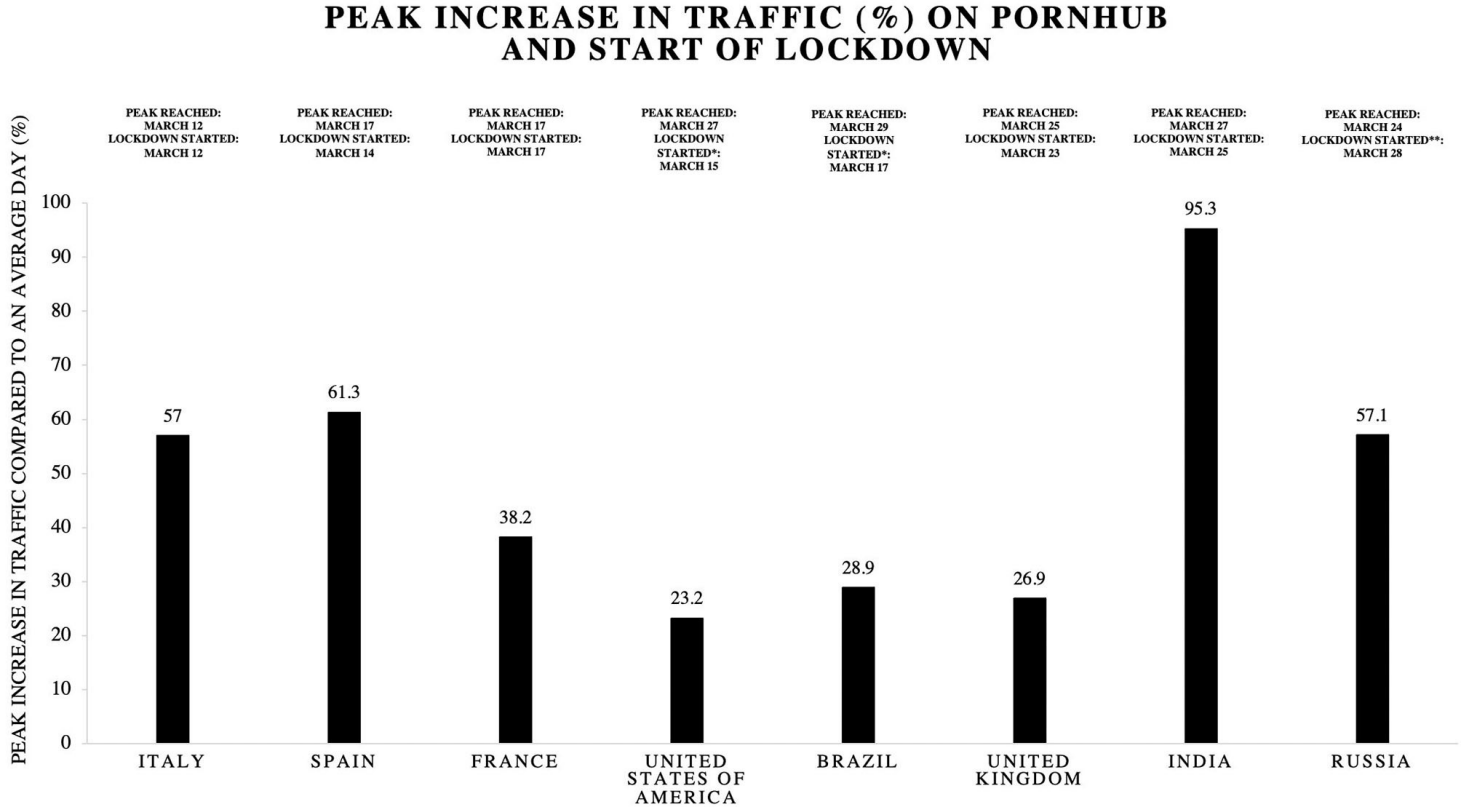
Peak Increase in Traffic compared to an average day (before the pandemic) on Pornhub^Ⓒ^ during COVID-19 Pandemic with Starting Date of Lockdown and Date of Peak Increase in Traffic in Selected Countries. This figure has been generated by the authors of this review based on data from Pomhub Insights (data from observations in the period from February 24 to March 17, 2020, retrieved from: https://www.pornhub.com/insights/corona-virus) and BBC News (data from observations in the period from January 15 to April 1, 2020, retrieved from: https://www.bbc.com/news/world-52103747). *Date of lockdown unclear **Localized lockdowns started earlier (date here refers to nationwide lockdown).

To put the 2 timelines (lockdown and rise in pornographic websites' traffic) relative to each other, Figure 1 presents the peak percentage change of 8 countries, along with the date on which the peak was reached and the date when a major lockdown was instated.

It is relevant to discuss Cooper's “Triple-A Engine” model (44) based on accessibility, affordability, and anonymity and how these factors may have been impacted by the lockdown. Smartphones dramatically increased accessibility to online content, enticing some people, who otherwise might have not done it, to consume pornography (45). On March 17, 2020, Pornhub^Ⓒ^ announced free services for France on its Twitter^Ⓒ^ account, which was followed by the highest increase in traffic the same day. Italy and Spain were also offered free premium content from Pornhub^Ⓒ^, causing an enormous spike in user traffic. Affordability, even pre-COVID, was at an all-time high with most video-sharing websites allowing users to watch free content without any kind of financial commitment.

Cooper's concept of anonymity can be extrapolated to the idea of privacy as well. Due to the taboo nature of pornography in several cultures (46), individuals prefer anonymity online. This attraction to anonymity is also related to feelings of sexual freedom and expression (44). While some areas of India and most Islamic countries restrict access to pornography online due to social and/or religious reasons (47), laws regarding pornography vary widely across the world. Still, a ban/restriction can be circumvented due to the advent of virtual private networks (VPN), increasing accessibility and providing an additional layer of online anonymity. In fact, worldwide interest in VPNs on Google^Ⓒ^ has shown a peak on 17th March 2020, and countries that were hit the hardest by pandemic there have been up to a 160% increase in VPN usage between March 8 and 22 (48) (temporally associated with a rise in Pornhub^Ⓒ^ use, as shown in Figure 1). Furthermore, on August 28^th^, due to a technical error, Zoom^Ⓒ^ had stopped working from 8 a.m. to 2 p.m. (in the United Kingdom and East Coast of the United States), and a peak 6.8% increase in porn usage was noticed during that time' (42).

Döring explains how technology-mediated sexual contact, which was previously a relatively taboo subject, was now normalized, and sometimes even openly endorsed by authorities as the safer option compared to in-person sexual interactions. Pornography use, specifically, is considered positive and called a “constructive coping behavior” to overcome “boredom and fear” (49). Searches using the words ‘corona’ (18 million) or ‘quarantine’ (11 million) have also been notable on Pornhub^Ⓒ^. This is what some have termed “eroticization of fear” (50), but others feel that viewing aggressive pornographic content could potentially fuel an individual's abusive sexual tendencies (51). The COVID-19 pandemic has limited possibilities for casual sex and other behaviors, making individuals lean to pornography as the most accessible, affordable, and anonymous alternative (52). An intriguing risk-factor is described under “moral incongruence” and connected to religiosity and morality of an individual (53). It argues that a person will be at higher risk of developing an addiction to pornography due to the perceived misalignment with one's behaviors and one's beliefs (for example, religious). Even a “normal” duration spent on pornography can cause symptoms of pornography addiction (54) (distress and preoccupation) due to the conflicting behaviors and beliefs. Return to troubled families can also be a risk-factor during COVID-19, as dysfunctional or weak family relations have also been correlated with greater pornography use, particularly in adolescents (55).

Davis proposed that the combination of a “diathesis” (an underlying vulnerability) with a “stress” (such as the current pandemic and/or the lockdown) could prompt the development of a PUI (12), a proposition supported by other authors (56–58). This would place individuals with underlying psychopathology at greater risk. Studies have also proven an association of conditions like attention-deficit/hyperactivity disorder (ADHD) with increased risk of internet addiction (49). Underlying psychopathology may also cause an increase in porn consumption as a “compensation” method. “Forced abstinence” from an addictive behavior (like a period of inability to play an online game) has the potential to cause withdrawal, leading the individual to explore other ways to compensate and fill in the gaps (59), explaining how such behavior toward one medium can outgrow into others. A study from South Africa highlighted the possible “substitution” of an original addiction with new behaviors during periods of forced abstinence, specifically highlighting a case that used pornography as a substitute due to its easy attainability even during the lockdown (60).

Further, “escapism” is a relevant concept when analyzing the use of pornography by those suffering from body image issues. There is a presumed association with excessive internet (and pornography) use and body image avoidance (61) as individuals can control their image online and find this escape sexually liberating. It has been reported through a cross-sectional study (62) and explained through etiological models (12, 63, 64) that an association between social anxiety and internet addiction exists because individuals like their “ideal self” online (65) and prefer it over face-to-face communication.

## Prevention and Harm Reduction in the Post-Pandemic Era

Keeping in mind the current COVID-19 pandemic and the related restrictive and containment measures (e.g., the lockdown), addiction and mental health professionals should take into account not only the subsequent psychosocial burden, the emergence of new psychiatric onset (or relapse and/or worsening of preexisting psychopathologies) amongst the most vulnerable people, but also the tangible and concrete risk that the emergence of behavioral addictions has steeply risen. Local and international authorities have released guidelines to curb problematic internet use (66) and Table 1 adapts them to present suggestions specific to POPU.

**Table 1:** General and Specific guidance for curbing problematic online pornography use.

**General**	**Specific**
Scheduling daily time for physical activity to allow for “destressing” and raising dopamine levels	Creating an abstinence list detailing specific problematic behaviors with a specific plan for avoidance of or non-engagement in the identified behaviors
Engaging in other vocational activities like reading, writing, listening to music, etc	Focusing on mindfulness exercises to carefully observe habits, time spent on various activities, urges, etc
Enjoying social activities and maintaining relationships with family on a regular basis	Actively building trust with closest members in family, especially the significant other, and practicing healthy communication and transparency
Intentionally limiting daily screen time for outside work-related activities and using apps that provide reports about how much time was spent on online activities per week	Installing internet accountability software on digital devices
Keeping in touch with friends, relatives and acquaintances during times of physical distancing	Seeking out programs that might support individual recovery and foster a sense of accountability through a sponsor, e.g., Sex and Love Addicts Anonymous meetings

Pornography or internet addiction can make “re-adaptation” after the pandemic complicated and difficult to cope for individuals who have, owing to elongated periods of staying at home, adopted this lifestyle and have developed a dependence on these activities as an essential part of their lives (67). Some articles have warned about pornography consumption normalizing violence against women and potentially leading people to engage in it in real life during the lockdown when women are alone with men in the house (68). Döring therefore stresses on target-specific sex education, especially for adolescents, to avoid any negative outcome (49). While many recommendations for treatment plans of internet addiction and IPA have been published, they essentially revolve around supporting the individual's needs, controlling damage to and rehabilitating interpersonal relationships, and preventing relapse (69).

Pharmacological interventions with different drugs like naltrexone (22) or quetiapine with citalopram (70) have been examined. Paroxetine has been used to treat IPA and has shown partial efficacy (71). Psychological treatments have acted as a key tool in treating addictions. Showing positive results for internet addiction in 2013 (72), cognitive-behavioral therapy (CBT), which lasts for 12 weeks and has a 6-month follow up, has been one of the most-studied psychological therapy used for behavioral addictions (73, 74). Another 12-week model is the acceptance and commitment therapy (ACT) (75), shown to be effective in IPA. Twelve-step treatment programs have been historically successful in tackling addictions by also significantly reducing comorbidities like depression. It is however suggested that a combination of both pharmacological and psychological is essential to effectively tackle addiction (76). Brand et al. suggests that combined intervention to target the mediating and moderating factors (in the I-PACE model explaining the development) of such behaviors as predisposing vulnerability (genetic or neurobiological) usually remains unaffected (38). In 2014, Brand et al. highlighted the importance of evaluating patients' coping style for effective treatment and recovery (77). In the COVID-19 era and beyond, employing telepsychiatry with online support groups is possibly going to prove beneficial (78).

Greater awareness of the potential risks during the lockdown can help break the stereotype of behavioral addictions and encourage seeking help from competent professionals. Realizing that such behaviors potentially affect the community as a whole can help in prevention by means of more thorough guidelines and easy-to-access information.

As opposed to many substances of abuse, the object and means of behavioral addictions, including the internet, are ubiquitous in daily life and hard to avoid; they are even needed. Prevention of first exposure to the internet, and then complete abstinence from the internet for people already using it seems particularly unrealistic. Thus, primary prevention of PUI and rehabilitation of individuals with internet-related psychopathology will usually require the integration of internet use into a healthy lifestyle, having its own place and priorities within the personal, professional, and relational goals and duties of each individual.

Table 1 offers specific and general guidance for prevention and alleviation of problematic online pornography use; most of the points presented there are valid for PUI in general. These include the incorporation of healthy physical routines and leisure activities as alternatives or replacements of pornography, the maintenance of meaningful social relationships, monitoring screen time, and seeking specific help when needed.

## Conclusion

Problematic internet and online pornography use have been reported to constitute an increasing burden in public mental health since the 2000s, yet psychopathological models and diagnostic criteria have lacked consensus, and the body of evidence on the effectiveness of therapeutic approaches is still in scarce. The COVID-19 pandemic has forced millions indoors and needed of the mediation of screens to work, maintain social interactions, and carry out everyday activities such as shopping; this has exposed many to a higher risk of developing or worsening problematic use of internet and pornography.

The current pandemic and its aftermath represent a challenge and an opportunity to revisit the conceptual discussions on these internet-mediated problems and to advance etiological and epidemiological research, agree on diagnostic criteria, and identify effective interventions to better understand and minimize the individual and social impact of these. We hope our review provides an up-to-date perspective on the topic and guidance to start addressing the problems of pathological internet and online pornography use.

## Author Contributions

AA and IU conceived the original idea and designed the outlines of the study. HA, AA, MD, IU, VP-S, and SV wrote the draft of the manuscript. HA, AA, MD, and IU prepared the figures of the manuscript. VP-S, RRam, LO, RF, MO, RRan, AV, and SV performed the literature review and improved the manuscript. All authors contributed to the article and approved the submitted version.

## Conflict of Interest

The authors declare that the research was conducted in the absence of any commercial or financial relationships that could be construed as a potential conflict of interest.
